# Improving the engine power of a catalytic Janus-sphere micromotor by roughening its surface

**DOI:** 10.1038/s41598-018-22917-2

**Published:** 2018-03-15

**Authors:** Brooke W. Longbottom, Stefan A. F. Bon

**Affiliations:** Department of Chemistry, University of Warwick, Gibbet Hill Road, Coventry, CV4 7AL U.K.

## Abstract

Microspheres with catalytic caps have become a popular model system for studying self-propelled colloids. Existing experimental studies involve predominantly “smooth” particle surfaces. In this study we determine the effect of irregular surface deformations on the propulsive mechanism with a particular focus on speed. The particle surfaces of polymer microspheres were deformed prior to depositing a layer of platinum which resulted in the formation of nanoscopic pillars of catalyst. Self-propulsion was induced upon exposure of the micromotors to hydrogen peroxide, whilst they were dispersed in water. The topological surface features were shown to boost speed (~2×) when the underlying deformations are small (nanoscale), whilst large deformations afforded little difference despite a substantial apparent catalytic surface area. Colloids with deformed surfaces were more likely to display a mixture of rotational and translational propulsion than their “smooth” counterparts.

## Introduction

Small particles dispersed in a fluid that undergo continuous energy consumption to induce motion are an interesting form of active matter^1^. Within this branch of colloid science a particular focus on self-propelling particles has emerged^2–9^. These “micromotors” are under the microscope (quite literally) for their potential in a variety of scientific pursuits, examples include drug delivery^10^, medical applications^11^, environmental remediation^12^ and the popular studies of dissipative assembly (swarming)^13^.

**Figure 1:**
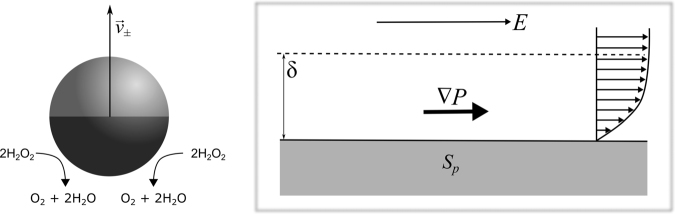
Schematic of self-generated phoresis. Decomposition of hydrogen peroxide occurs asymmetrically at the Pt cap (dark patch) of the PS-Pt microsphere leading to propulsion with velocity, $\mathop{{v}_{\pm }}\limits^{\longrightarrow}$, the direction of which depends on the local chemical environment. The interaction of an external or self-induced field, *E*, with the surface of the particle, *S*_*p*_, (in the interfacial region, δ) generates a tangential pressure gradient,∇*P*, which in turn drives fluid flow.

A prominent model system in self-propulsion studies are poly(styrene) (PS) microspheres with a catalytic platinum (Pt) cap surface coated on part of the sphere (so-called Janus PS-Pt “swimmers”) dispersed in water containing hydrogen peroxide (H_2_O_2_) as fuel^7^. Propulsion is facilitated by the decomposition of H_2_O_2_ to oxygen (O_2_) and water (H_2_O) on the Pt surface. Theory predicts that spherical particles with a catalytic patch should move *via* a self-generated phoretic mechanism when placed in a homogeneous medium containing fuel^14^. Phoretic transport is classically described as the colloidal motion induced by the interaction of external fields with the interfacial boundary region of the particle^15^. The external field can consist of a solute concentration gradient which here is established by the catalytic chemical reaction on the Pt-coated region of the surface of the particle. In this way the particle is said to be autonomous and “self-phoretic”. For PS-Pt Janus particles it was originally thought to occur purely by self-diffusiophoresis, whereby a concentration gradient is established, dynamically, in the direct environment of the microsphere through the catalytic reaction at its surface. The precise mechanism has been the subject of debate due to observations of ionic effects, including influence of propulsion direction, indicating electrophoretic contributions to the motion^16–18^. Electrophoresis infers spatial localised variations in the diffusive electric double layer around the microsphere. Nevertheless, in both cases the propulsion originates from a local pressure imbalance resulting in the generation of an effective slip velocity along a thin interfacial layer driving fluid flow (Fig. 1).

Self-generated phoretic motion of PS-Pt Janus microspheres leads to velocities on the order of 2−17 μm·s^−1^ ^16,19^. Which parameters influence the velocity? Two fundamental design properties of the colloidal object, proposed in the original theoretical framework for phoretic propulsion^20^ and recently highlighted by Michelin and Lauga^21^, govern speed: 1. Surface chemical activity which involves the generation of the local gradient by chemical reaction and 2. Surface phoretic mobility, which concerns the generation of fluid flow from a local gradient. In other words we must consider how fast a concentration gradient can be established and how effectively the surface interacts with this gradient to generate flow. Considering our system for property 1: the surface coverage of Pt catalyst, its topography (roughness), and thickness will determine the overall catalytic activity and hence influence the magnitude and shape of the induced chemical gradient. With respect to property 2, our geometry on the micron-scale is fixed. However, nanoscale roughness of our catalytic patch may influence the interaction at the slip boundary.

To date there are a few studies that examine the effect of surface topography on the self-propulsion of micromotors^22–25^. Choudhury *et al*. reported a speed increase of up to four times for SiO_2_-Pt Janus microspheres with a rough Pt surface compared to their smooth counterparts^24^ attributed to the increase in catalytic surface area. Nanoscale surface roughness was imparted by first depositing an 80 nm SiO_2_ under layer and a subsequent thin (8 nm) Ti layer for adhesion of Pt at different substrate angles using a glancing angle deposition (GLAD) method. A GLAD method was also utilized by Archer and coworkers to synthesize PS-Pt micromotors with defined rotational velocity^26^.

Here, we introduce a methodology to access PS-Pt micromotors with irregular surface morphologies and investigate their propulsive behaviour. The self-propelling colloids are made from spherical PS precursors which are roughened using a recently established physical route^27^, prior to Pt deposition by sputtering. With this strategy the underlying surface chemistry should be practically identical in each case and we can make reasonable conclusions about the propulsive mechanism and magnitude.

## Results

### Fabrication

**Figure 2:**
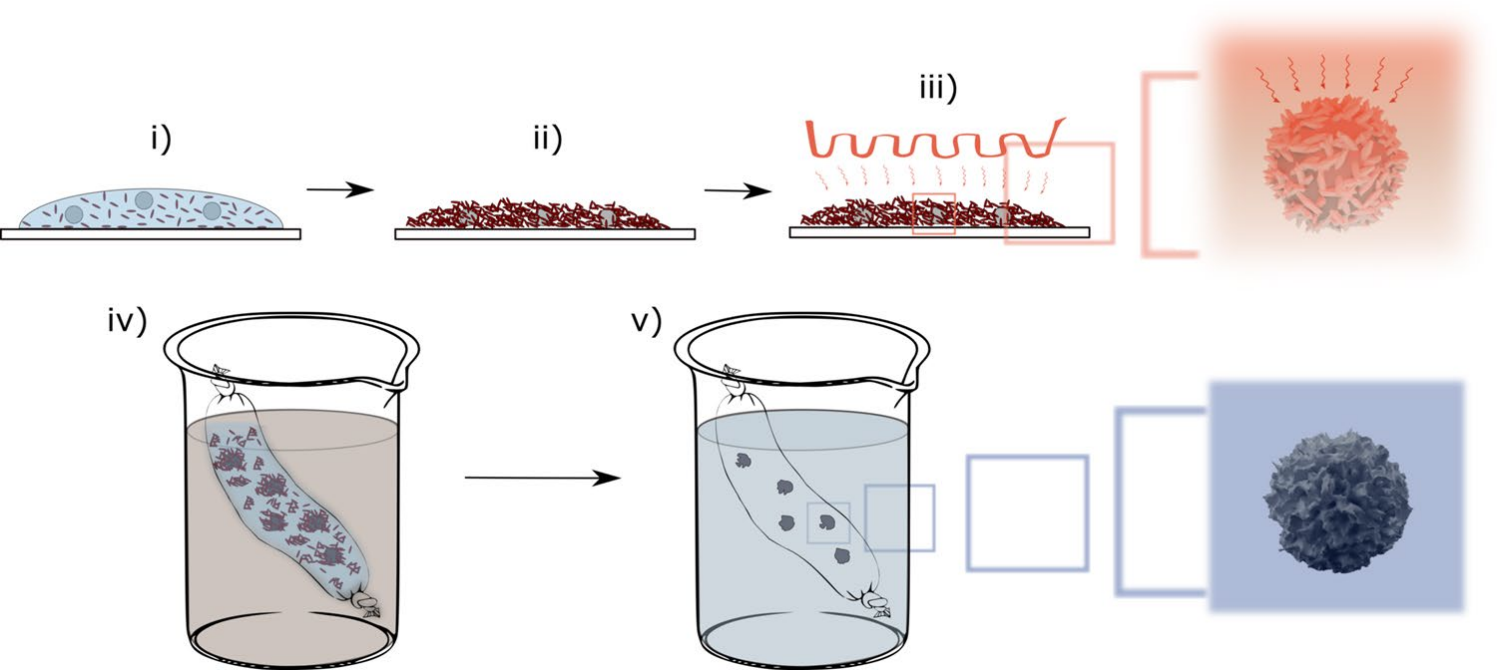
Illustration of the fabrication procedure for roughened poly(styrene) microparticles. A water-based dispersion consisting of 0.1 wt. % PS spheres and 10 wt. % inorganic particles of a bespoke shape (being calcium carbonate or zinc oxide) was drop-casted to a glass slide (**i**), time was allowed to dry at ambient temperature (**ii**), the slide was transferred to an oven at 110 °C or 130 °C for t = 120 mins and subsequently allowed to cool to ambient temperature (**iii**), The particles were removed from the glass surface, re-dispersed in water, and dialysed against 2.0 molar acetic acid (*aq*.) to dissolve the inorganic particles (**iv**), after dissolution of the inorganic colloid the isolated roughened PS dispersion was purified further by dialysis against de-ionised water (**v**).

**Figure 3:**
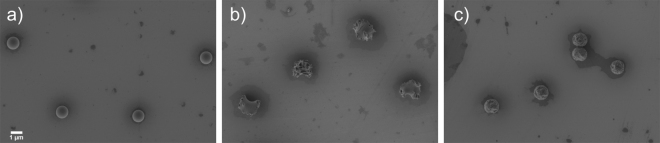
Scanning Electron Microscopy (SEM) images of poly(styrene-*co*-methacrylic acid) microspheres synthesized by dispersion polymerisation (**a**), after deformation at 130 °C in the presence of cigar-shaped calcium carbonate to impart “large deformations (*ld*)” (**b**) and at 110 °C with spherical zinc oxide to yield “small deformations (*sd*)” (**c**).

In order to fabricate Janus PS-Pt microspheres with varying surface deformations, PS microspheres synthesized by dispersion polymerisation were first roughened using a previously established procedure (see Fig. 2)^27^. In a typical dispersion polymerisation, a solution of styrene (12.15 g) in ethanol (86.25 g) is polymerized by free radical polymerisation using azobisisobutyronitrile (AIBN, 0.1215 g) as initiator, and in presence of poly(vinyl pyrrolidone) (PVP K-30), 0.85 g as colloidal stabilizer. Note that a small amount of methacrylic acid (MAA, 0.1215 g) was used as co-monomer to promote metal adhesion. The PS microspheres had a monomodal particles size distribution with an average diameter of 1.19 ± 0.02 μm (see Fig. 3a for a representative SEM image).

The PS microspheres were deformed into their rough analogues by using a physical method which relies on capillary imbibition into an inorganic template at temperatures above the glass transition temperature of the polystyrene (see Fig. 2, and Fig. 3b and c for representative SEM images of the roughened particles).

Three distinct candidate samples were selected to be turned into micromotors based on the scale of the particle surface deformations (See Fig. 3 for SEM images). We refer to these PS particles as “smooth (*s*)”, having “large deformations (*ld*)” or “small deformations (*sd*)”. The first sample, a control standard, had no deformations applied and thus exhibited a “smooth” surface (Fig. 3a). The second sample was deformed above (130 °C) the glass transition temperature (T_g_) of PS, in the presence of cigar-shaped calcium carbonate with d ~ 0.2 μm, l ~ 0.75 μm (Fig. 3b). The PS particle shows “large deformations” characterized by deep irregular craters across the entire surface of the particle with an overall slight departure from a spherical shape. A third and final candidate selected was deformed in the presence of small (d ~ 0.1 μm) oblong-shaped zinc oxide (ZnO) nanoparticles at 110 °C (Fig. 3c). Nanoscale roughness, and hence “small deformations”, with similar dimensions to the ZnO was imparted to the PS microsphere. As a side note, in our previous study it was found that PS microspheres deformed in the presence of ZnO had buckled structures which was not found in the current work. We suggest this could be down to a change in surface chemistry due to presence of methacrylic acid groups.

**Figure 4:**
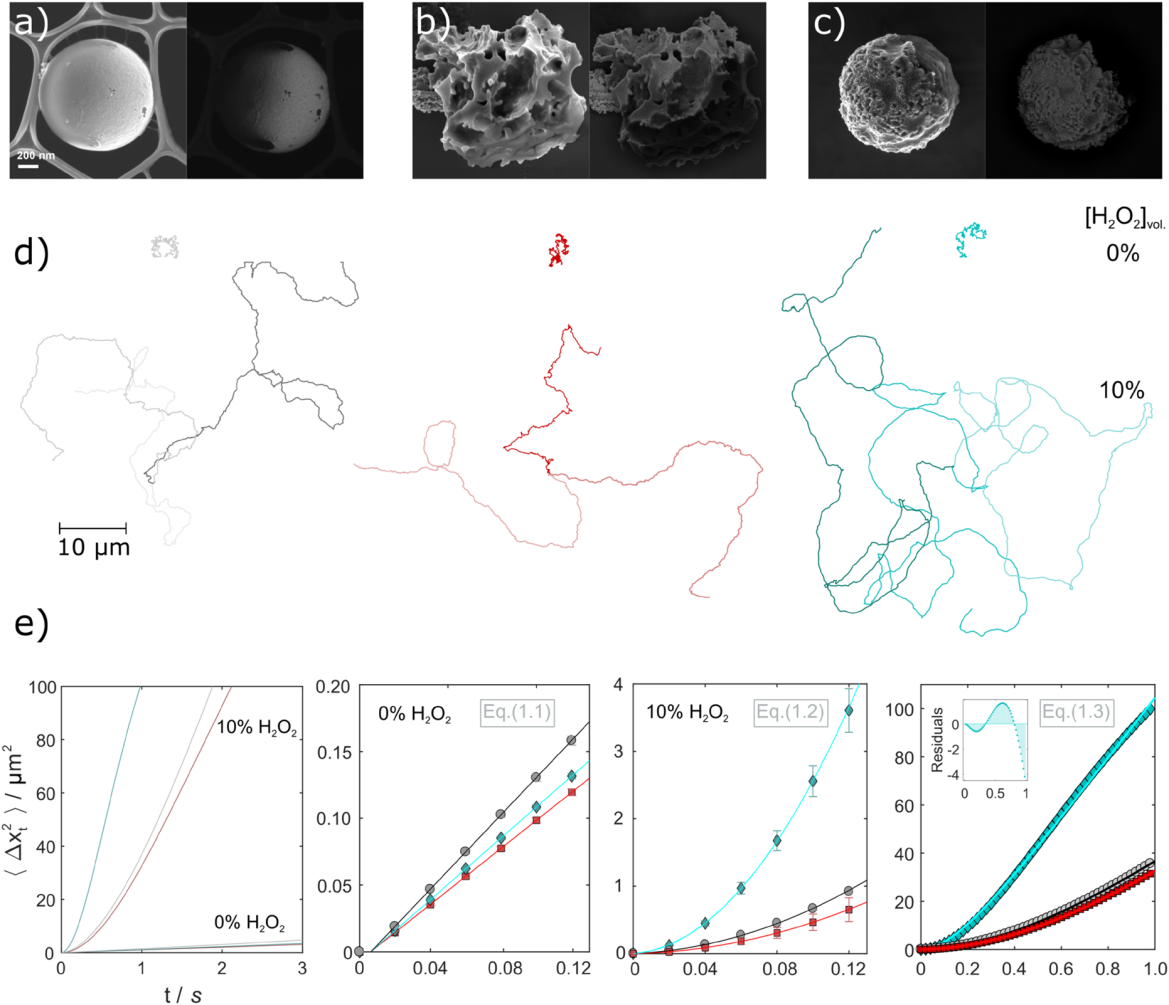
Scanning Electron Microscopy (SEM) images of poly(styrene-*co*-methacrylic acid) microspheres half-coated with platinum (**a**–**c**) with large surface deformations (**b**) and small surface deformations (**c**). Scale bar = 200 nm. The left images are from the detection of secondary electrons, right are primarily from back-scattered electrons. Representative particle trajectories (10 s) with no fuel present and at 10 vol. % H_2_O_2_ (**d**). Average mean-squared-displacement (MSD),$\,\langle \Delta {x}_{t}^{2}\rangle$, graphs at given delay time, *t* (**e**) for “smooth” PS-Pt micromotors (grey), with large surface deformations (*ld*) (red) and small surface deformations (*sd*) (cyan) with no fuel present and at 10 vol. % H_2_O_2_. Fitting regions are displayed for determination of D at 0% fuel, which was used to constrain fits for determining *v* and *τ*_*R*_ at 10% fuel. Fitting equations are labelled in accordance with the main text and an inset of the residuals for the weighted fit of Eq. (1.3) to the (*sd*) MSD is included (bottom right).

Particle dispersions (volume fraction, *φ* ~ 0.01 in iso-propyl alcohol) for each candidate were prepared to deposit sub-monolayers of particles onto glass slides. Subsequently physical vapour deposition (PVD), specifically sputtering, of Pt (~15 nm) onto deposited PS particles resulted in the desired half-coated Janus PS-Pt morphology (Fig. 4a–c). Note again that we found it essential to incorporate a small amount of carboxylic acid-functional co-monomer, methacrylic acid, in the dispersion polymerisation in order to facilitate adhesion of Pt onto the particle surface. Without the co-monomer present, the Pt film flaked off upon attempted re-dispersion of the microspheres. To help prevent re-dispersion of Pt flakes and nanoparticles, wet lens tissue was gently wiped over the glass slide to lift off only the PS-Pt Janus micro particles.

### Propulsion Experiments

To begin, the translational Brownian diffusion of each candidate sample was determined by 2D particle tracking under aqueous conditions at 25 °C and without fuel present. Particles were tracked near the underlying glass surface as they tend to sediment here due to a density mismatch between the poly(styrene) microspheres and water, added to by the presence of the heavy Pt cap^28^. For 2D translational Brownian motion any boundary effects from the underlying glass surface are small^27,29^ and the significant mass anisotropy of the Pt cap does not have a large effect (Note: it does have a marked effect in 1D)^28,30^. Applying linear fits to the mean-squared-displacement (MSD), $\langle {\rm{\Delta }}{x}_{t}^{2}\rangle$, (Fig. 4e) yields the 2D diffusion coefficient, *D*, according to Equation (1.1).

1.1 $$\langle {\rm{\Delta }}{x}_{t}^{2}\rangle =4Dt$$

Each particles MSD was fitted from *n* = 2 (0.02 s) to *n* = 7 (0.12 s) data points and subsequently an average value of *D* yielded: “smooth” *D*_*s*_ = 0.348 ± 0.022, large deformations *D*_*ld*_ = 0.263 ± 0.016 and small deformations *D*_*sd*_ = 0.289 ± 0.0156 μm^2^s^−1^. All are comparable to the theoretical values (*D* = 0.27 to 0.41 μm^2^s^−1^) from the Stokes-Einstein-Sutherland (S-E-S) relation, *D* = *k*_*B*_*T*/6*πηr*, for colloids of this size range (r = 0.6 to 0.9 μm). The differences between these *D* values is down to a change in the effective hydrodynamic radii of the deformed particles, in that the more deformed the particle is the larger its effective diameter (measured from the two furthest peripheral points) and therefore the lower *D* is, as shown previously^27^.

When 10 vol. % hydrogen peroxide (“fuel”) is present in the water phase, its decomposition into water and oxygen catalysed by the platinum coated particles induces propulsion with a translational velocity, *v*, that, far enough below the rotational diffusion time of the colloid, *τ*_*R*_, relates to the MSD according to Equation (1.2)^7^.

1.2 $$\langle {\rm{\Delta }}{x}_{t}^{2}\rangle =4Dt+{v}^{2}{t}^{2}$$

The theoretical rotational diffusion can be calculated from *τ*_*R*_ = 8*πηr*^3^/*k*_*B*_*T* which gives a minimum estimate *τ*_*R*_ ~ 1.5 s for our colloids. Thus from a quadratic fit of the MSD data at 10% of *τ*_*R*_ *i.e. t* = 0.15 s, *n* = 7 data points, *v* can be extracted^19^. Two fits was carried out on the data, one being unconstrained, the other constrained to the experimentally determined values of *D* from the Brownian results at 0% H_2_O_2_ (Table 1), corrected for a viscosity change caused by the introduction of 10 vol% hydrogen peroxide to the water (a minor effect). These constraints are valid as *D* and *v* are decoupled and *D* has little to no dependence on hydrogen peroxide concentration.

**Table 1:** Comparison of the determined diffusion coefficeints, *D*, and velocities, *v*, extracted from the parabolic fits, Equation (1.2), of mean-squared-displacements of PS-Pt micromotors (Fig. 4).

		*D*/µm^2^s^−1^	*v*/µm·s^−1^
smooth (*s*)	U	0.27	7.48
	C	0.35	7.24
rough *(ld)*	U	0.14	6.41
	C	0.26	5.99
rough *(sd)*	U	0.44	15.5
	C	0.29	15.59

U – unconstrained fits, C − fits were constrained to experimentally determined values of *D* at 0 vol. % Fuel.

PS-Pt micromotors with large surface deformations exhibited similar velocities to their “smooth” counterparts whereas those with small scale deformations saw an effective doubling of speed. In order to rule out additional effects of convective transport, which can erroneously raise *v*, the full expression for the MSD at all delay times^7^ was fitted to the data up to *t* = 1 s (Equation 1.3).

1.3 $$\langle {\rm{\Delta }}{x}_{t}^{2}\rangle =4Dt+\frac{{v}^{2}{\tau }_{R}^{2}}{2}[\frac{2t}{{\tau }_{R}}+{e}^{-2t/{\tau }_{R}}-1]$$

As the delay time, *t*, approaches *τ*_*R*_ the MSD begins to decay from a parabola back to a linear form as the direction of propulsion has been allowed time to fully randomise. The data shows good agreement with this expected behaviour when a constrained (using *D*) fit was applied (Fig. 4e). Further extraction of both *v* and *τ*_*R*_ for each particle is possible with this fit (Table 2).

**Table 2:** Comparison of the diffusion coefficient, *D*, velocities, *v*, and rotational times, *τ*_*R*_, determined by fitting Equation (1.3) to the mean-squared-displacements of PS-Pt micromotors up to *t* = 1 s (Fig. 4).

		*D*/µm^2^s^−1^	*v*/µm·s^−1^	*τ*_*R*_/s
smooth *(s)*	U	0.59	7.03	1.68
	C	0.35	7.36	1.41
	w	0.35	7.26	1.55
rough *(ld)*	U	4.8 × 10^−10^	6.75	1.83
	C	0.26	6.46	2.24
	w	0.26	6.24	3.01
rough *(sd)*	U	3.6 × 10^−8^	19.62	0.32
	C	0.29	19.40	0.33
	w	0.29	16.84	0.52

U – unconstrained fits, C – fits were constrained to experimentally determined values of *D* at 0 vol. % Fuel, w – weighted (1/*σ*^2^), constrained fits.

The velocities are comparable to those extracted from the parabolic fit (Eq. 1.2, Table 1) with a noticeable difference (~20%) only in the case of particles with small deformations (see Table 2). Rotation times, *τ*_*R*_, are also comparable with theory for the PS-Pt micromotors with “smooth” and large deformations but significantly lower than expected for those with small deformations assuming a translational propulsion mechanism. Note that an inherent issue with unconstrained multiple parameter fits is that they can force unphysical parameter values to materialize, such as the exceedingly low values extracted here for *D* of the deformed PS-Pt micromotors. Also the error in MSD data scales with the square and thus fitting to long delay times introduces significant error to the analysis. Weighting the fits with weights equal to the reciprocal of the standard deviation squared, *w* = *1/σ*^2^, provides a more rigorous fit. Even in doing so we still extract a low value for *τ*_*R*_ for micromotors with small deformations suggesting more complex propulsion behaviour in this case.

An experimental route to minimize the error in MSD data involves tracking the particles for a longer overall time period (at least an order of magnitude of the fitting region). However in practice this is difficult as the rapidly moving micromotors can leave the field of view during imaging. Reducing the objective magnification, allowing for a larger field of view, to combat this lowers the resolution and thus increases particle localization error. Therefore the preferred method is by fitting Equation (1.2) to the early MSD data (Table 1) providing that the propulsion mechanism has first been distinguished from convection or other transport mechanisms^31^.

Upon close examination of the MSD for PS-Pt micromotors with small deformations a departure from the expected behaviour was noticed (Fig. 4e) with a tailing off of the parabola between 0.4 < *t* < 1.0 s, this is exemplified by a plot of the residuals (inset Fig. 4e). The change in MSD suggests a different propulsive mechanism may be present. A superimposed angular component to the velocity could potentially yield an MSD shape akin to this, as has been previously shown for PS-Pt micromotors with defined Pt cap size^26^. Using the expression proposed in this work appears to over fit our data if unconstrained, yielding an excessively high value for *D*, and reduces to a parabola with *v* = 9.66 µm·s^−1^ if constrained to more reasonable values of *D* and *D*_*r*_. Hence we can conclude that the effect is too subtle to probe with this analysis.

**Figure 5:**
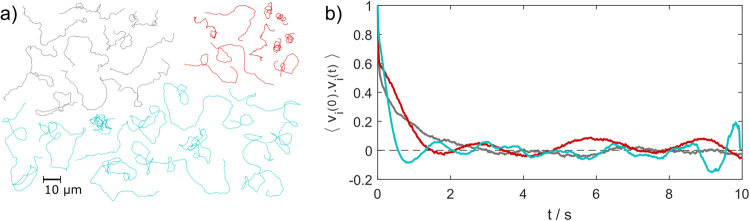
Propelled PS-Pt particle trajectories (10 vol. % H_2_O_2_) (**a**). Normalized velocity autocorrelation over 10 s (**b**) for “smooth” PS-Pt micromotors in 10 vol. % H_2_O_2_ (grey) and those with large deformations (*ld*) (red) and small deformations (*sd*) (cyan).

The normalized velocity autocorrelation (VACF), $\langle {v}_{i}(0).{v}_{i}(t)\rangle =\frac{1}{N}{\sum }_{i=0}^{N}({v}_{i}({t}_{0}).{v}_{i}({t}_{0}+n{\rm{\Delta }}t))$, reveals additional clues to the type of motion (Fig. 5). For a typical PS-Pt micromotor exhibiting translational propulsion this should decay to 0 after the rotational diffusion time, *i.e*. once the propulsive direction of the motor has been randomized. This is the case for our smooth PS-Pt micromotors, however for those with surface deformations there appears to be a small persistent oscillation (larger than the magnitude of noise) after the initial decay period. Again this is further evidence of some angular velocity, although the effect is fairly weak. Additional inspection of all particle trajectories in the sample revealed that some of the deformed micromotors followed a spiralling path for at least part of their trajectory (Fig. 5a).

The origin of angular velocity in the deformed PS-Pt micromotors is most likely caused by the irregularity of surface roughness. This can give rise to uneven catalytic activity across the surface of the particle, mass anisotropy and potentially enhanced interaction between the particle and glass surfaces. In a few cases we observed that PS-Pt micromotors with large surface deformations displayed predominantly translational propulsion when away from (or parallel to) the glass surface and began propelling rotationally suddenly. We hypothesize that this may be caused by an (unidentified) object or surface in close proximity (Supplementary Video S1). This behaviour was exclusive to this particle system and not observed for the “smooth” PS-Pt micromotors or those with small deformations. Note particles were only located in x and y. Precise z (height) locations would give more information about when this switch over in propulsion mechanism occurs. A combination of gravitational and hydrodynamic forces are likely to promote this phenomenon, as predicted and experimentally observed for self-phoretic micromotors near boundaries^32–35^.

### Microscopic Characterization – Resolving surface details

**Figure 6:**
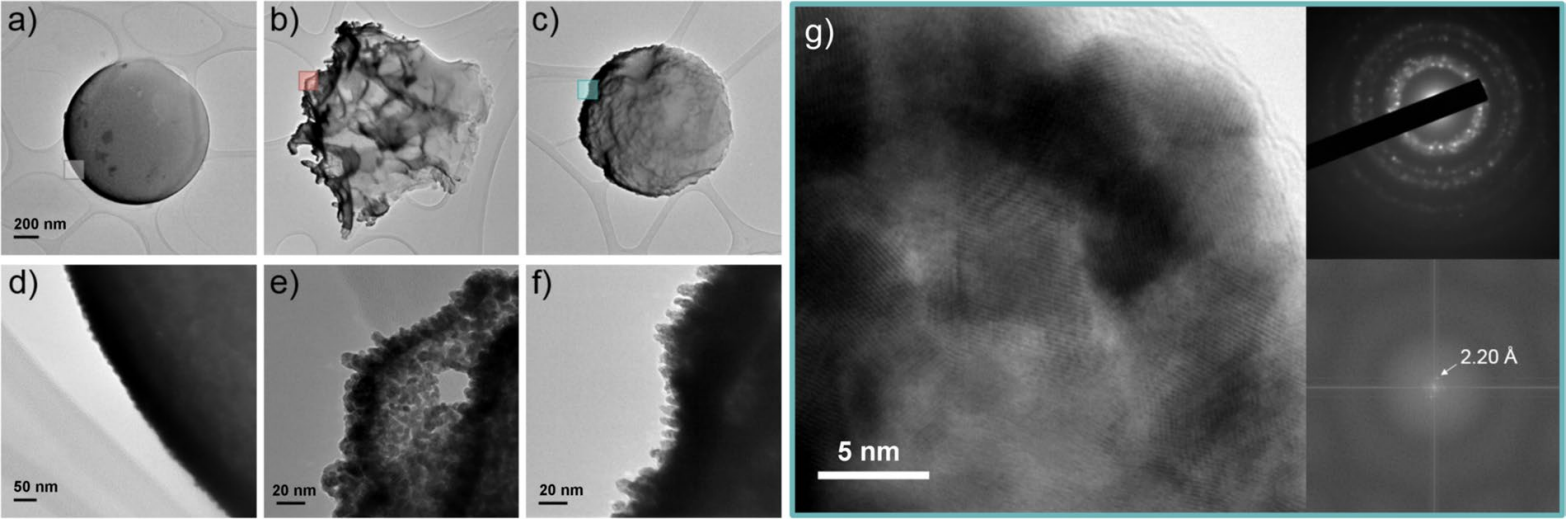
Transmission Electron Microscopy (TEM) images of poly(styrene-*co*-methacrylic acid) microspheres half-coated with platinum (**a**–**f**). Low (**a**–**c**) and high (**d**–**f**) magnification images of PS-Pt Janus micromotors without roughening applied (**a,d**), with large surface deformations (**b**,**e**) and small surface deformations (**c**,**f**). Scale bars (**a–c**) = 200 nm, (**d–f**) = 20 nm. Boxes highlight region of interest for high magnification images. High Resolution Transmission Electron Microscopy (HRTEM) image of a thin section of Pt sputtered surface of PS microspheres with small surface deformations (**g**). Selected area electron diffraction (SAED) pattern of the imaged region (top right) and fast fourier transform (FFT) of the central region of the image. The distance between lattice fringes, as measured from the FFT, is consistent with Pt (111). Scale bar (**g**) = 5 nm.

**Figure 7:**
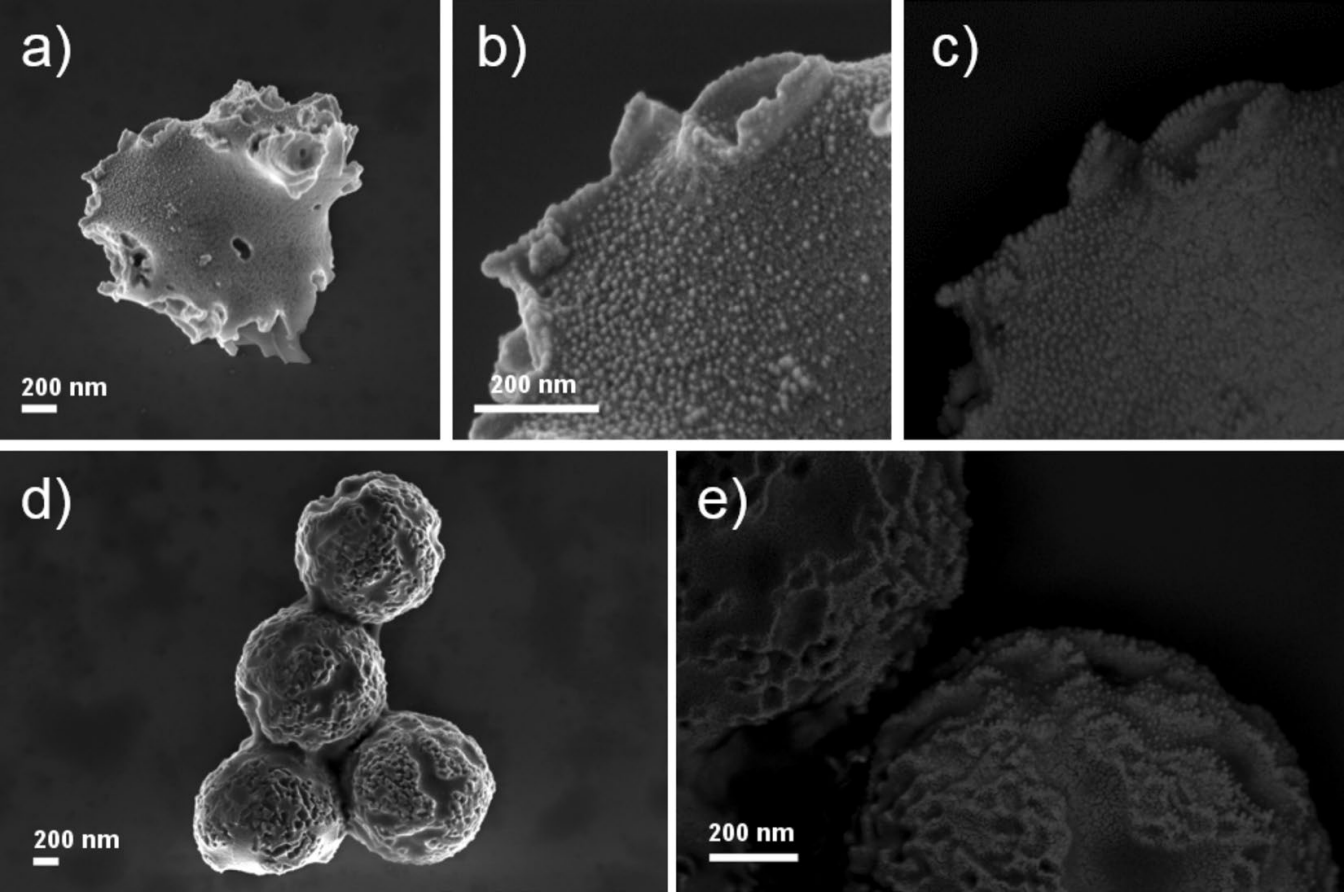
Scanning Electron Microscopy (SEM) images of deformed PS microparticles after sputtering with Pt (**a**–**e**). Back-scattered electron images (**c**,**e**) enhance compositional contrast and highlight locations of Pt pillars. Scale bars = 200 nm.

Before drawing conclusions from the motion analysis of deformed PS-Pt micromotors, it was imperative that the surface details of the PS-Pt micromotors were properly resolved. Transmission Electron Microscopy (TEM) images (Fig. 6) revealed the morphology of the supposed thin-film of Pt produced from the sputtering procedure. The un-deformed PS-Pt particle appeared to have a largely “smooth” surface with features on the order of 1−2 nm, in contrast to the deformed particles upon which pillars of Pt (~10 nm) were found. The presence of these features is intriguing considering the underlying surface chemistry and sputtering procedure was unchanged. Upon close examination it appears that areas of high curvature, *i.e*. sharp edges, could promote their formation (SEM, Fig. 7). This may be due to low adatom diffusion and effective “pinning” of Pt nuclei on these surface asperities during physical vapour deposition. Columnar features with high porosity are proposed to be formed at lower deposition temperatures^36^ when surface diffusion is limited. High resolution TEM and selected area electron diffraction (SAED) (Fig. 6g) of the Pt features on the surface of particles with small deformations reveals a polycrystalline structure with a predominant lattice spacing of 2.20 Å indicative of Pt (111)^37^. It would be interesting to properly resolve the exposed faces of the Pt pillars and thus the active sites of the catalst^38^, however the large underlying insulating polymer material of the microsphere makes this a significant challenge. Nevertheless we can conclude that these features lead to a vastly increased catalytic surface area which in turn affects the overall reaction rate at the surface.

## Discussion

Sputtered metallic thin films on an underlying surface universally exhibit a certain roughness^39^. It is logical that this roughness is a function of the amount of material deposited and that also the wetting/adhesion characteristics of the underlying substrate play a role^40^. Indeed Choudhury *et al*. varied surface roughness on spherical silica micromotors by depositing a significant (80 nm) underlayer of silica before sputtering 20 nm of Pt^24^. In this current study we have shown that relatively small changes in the polymer particle surface roughness can promote the formation of nanoscopic Pt pillars. These columnar features further increase the fractal dimension of surface roughness and hence there is a larger than expected catalytic surface area. As a direct result, there will be a shift in velocity magnitude for the composed micromotor. Therefore we suggest that a keen eye (and high resolution electron microscope) are crucial for experimental studies of micromotors fabricated in this manner.

For most Pt micromotor studies, homogeneous catalytic activity is assumed across the catalyst surface. In reality, varying thickness of the catalyst layer^17^ and crystallographic orientation affect the number distribution of exposed active sites. The magnitude of velocity is therefore further dependent on these properties.

**Figure 8:**
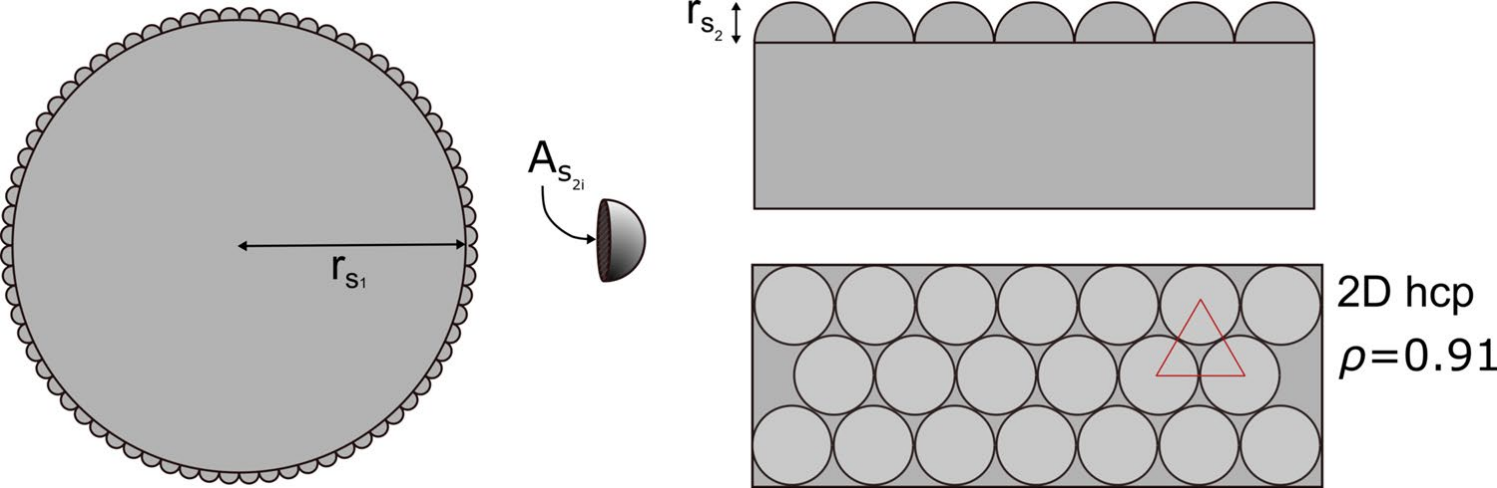
Packing of small hard hemi-spheres, s_2_, onto a larger sphere, s_1_, as a model for roughness.

As a back-of-the-envelope thought experiment, if we consider a surface roughness to be the product of depositing small hemi-spheres on a large sphere (Fig. 8), the maximum increase in surface area is less than two times the original sphere’s surface area.

Assuming 2D hexagonal close-packing (face-centered-cubic, fcc) of s_2_ on s_1_ to give a packing efficiency, *ρ* = 0.91. The new surface area of the rough sphere *A*_*rs*_ can be approximated by dividing the area covered by hemispheres, *ρA*_*s*1_, by the intersecting face of each hemisphere, *A*_*s*2*i*_, to yield the number hemispheres covering the surface. The product of this and the area of each exposed hemisphere, *A*_*s*2_, plus the uncovered surface gives the new, rough surface:

1.4 $${A}_{rs}=(\frac{\,\rho {A}_{s1}}{{A}_{s2i}}){A}_{s2}+(1-\rho ){A}_{s1}$$

The relative increase in surface area is *A*_*rs*_/*A*_*s1*_ = 1.91 which in fact holds for any r_s2_ < r_s1_ in this model. Note that as r_s2_ increases relative to r_s1_, the curvature of the large sphere becomes more important and thus the intersection area increases. Also perfect fcc packing is not possible on a curved surface as the sum of the interior angles of the triangular lattice must exceed π, introducing strain and therefore defects^41^. These effects are omitted here for simplicity.

Real surface roughness is much more complex, however it stands without saying that in order to increase it, higher aspect ratio features or increased fractal dimension are necessary. Surface roughness may promote faster motion by both increased catalytic activity and a modification to the slip velocity - known to have a dependence on roughness^42^.

It was initially intriguing to us that micromotors with large surface deformations had a similar propulsion velocity to their non-deformed cousins as we expected a larger catalytic surface area to promote faster motion. Large surface deformations lead to an effective diameter increase of ~40% as found in a previous study^27^. If we consider no change in the phoretic mechanism and ignore effects of the altered particle shape, an equivalent reduction in propulsion velocity should be observed, $v\propto 1/r$^19^. Only a 17% reduction was measured, suggesting size alone cannot explain the result. We propose that the significant irregularity in the particle shape and presence of larger craters may lead to a hindrance in establishing an even concentration gradient and flow profile about the particle.

In conclusion, we have demonstrated that the surface roughness of micromotors influence their propulsion behaviour significantly. Surface deformations in our catalytic micromotors lead to considerable enhancement of the nanoscale surface roughness of the platinum catalytic layer, hereby increasing propulsion velocities and thus “engine power”. Moreover, torque-driven oscillations in the displacement of the micromotors was observed, with a questionable correlation between frequency of surface roughness and frequency of motional torque.

## Methods

### Materials

Styrene (≥99%) and poly(vinyl-pyrrolidone) (PVP K-30: Average Mw = 40,000 g mol^−1^), methacrylic acid (99%), azobisisobutyronitrile (AIBN) (98%) and dialysis tubing cellulose membrane (typical molecular weight cut-off = 14,000) were obtained from Sigma Aldrich. AIBN was recrystallized in methanol to remove impurities prior to use. Acetic acid glacial (>99%) and Ethanol p.a. were obtained from Fischer Scientific. Hydrogen peroxide (30 wt. %, *aq*.) was obtained from Honeywell. Calcium carbonate, Socal P3 powder (diameter = 250–350 nm) (length = 500 nm-1μm) was received as a donation from Akzo-Nobel. Calcium carbonate, PCC rods powder (diameter ~ 500 nm) (length = 3–15 μm) was received as a donation from Unilever. The Z-COTE zinc oxide (diameter ~ 120 nm) was received as a donation from BASF.

### Dispersion Polymerisation of micron-sized poly(styrene-co-methacrylic acid) spheres

AIBN (0.1215 g), PVP K-30 (0.85 g), ethanol (86.25 g), styrene (12.15 g) and methacrylic acid (MAA, 0.1215 g) were charged to a 250 mL round bottom flask and purged with nitrogen for 40 minutes. Next, the flask was submerged into an oil bath heated to 70 °C (IKA RCT Basic) and left to react for 24 hours whilst agitating with a magnetic stirrer and kept under a slight nitrogen gas overpressure. The obtained particle dispersions were centrifuged and re-dispersed in de-ionized water and ethanol three times each.

### Physical deformation of PS-MAA microspheres

Physical roughening was performed by heating samples above the glass transition temperature of poly(styrene) in the presence of a hard colloidal matrix for *ca*. 2 hr, as described previously^27^. In this study the oven temperature was 110 °C for those deformed with small ZnO particles and 130 °C for those deformed with CaCO_3_.

### Physical Vapour Deposition of thin films of platinum

Low volume fraction (φ = 0.01) suspensions of PS-MAA particles in isopropyl alcohol were deposited onto glass microscope slides. Sputtering was performed using a platinum source in a DC Magnetron sputter coater (Quorom Technologies SC7640) at 2.5 kV, ~20 mA for two consecutive 60 s cycles, ~15 nm film thickness according the manufacturer.

### Particle Characterization

Samples for SEM were prepared by dropping suspensions and allowing them to dry onto silicon wafers supported on aluminium stubs with a conductive copper adhesive. SEM imaging was performed on a Zeiss Gemini SEM 500 at 2 kV. Back scattered electron images were captured using the InLens detector with a bias of 450 V.

For TEM analysis samples were prepared by dropping a relatively dilute dispersion (~0.01 wt. %) of particles in water on to lacey carbon grids and allowing the samples to dry. Particles were imaged using a JEOL 2100.

### 2D Particle Tracking Experiments

Suspensions of PS-Pt particles were prepared by removing them from the glass slides by careful wiping with 1 × 1 cm^2^ wetted lens tissue. The lens tissue was then inserted into small Eppendorf tubes containing approximately 0.5 ml de-ionized water. Tubes were then vortexed to remove the PS-Pt particles from the lens tissue and suspend them in solution. For experiments when fuel was present, 50 μl of PS-Pt particle suspension was pipetted into 950 μl of 10 vol. % H_2_O_2_.

Two parallel lines of grease were drawn out to the approximate size of the cover slips being used (20 mm × 20 mm) onto a Linkam Warm Stage. The coverslip was placed lightly over the lines of grease and a micropipette was used to inject the sample solution underneath the assembled cell. Capillary action ensures the solution fills the well and it is subsequently sealed at the other edges with grease. Videos (20–30) of 5–10 particles in motion near to the underlying glass, 1024 × 1024 px. at 50 fps for 10 s, were recorded using an Andor Zyla 4.4 Plus camera attached to an Olympus IX73 inverted light microscope. Time was allowed for the Linkam warm stage to heat up to 25 °C. A magnification of 60× (Olympus LUCPlanFLN 60× Ph) was used with phase to enhance the contrast of particles.

A tracking algorithm, Trackmate^43^, distributed with imagej(Fiji) was then utilized to perform the 2D particle tracking. Our analysis was then performed using msdanalyzer, a Matlab class created for analyzing tracking data^44^. Fits were performed using the Matlab curve fitting toolbox.

## Electronic supplementary material

Supplementary Information

Supplementary Video S1
