# Supplementary material for: Improving the engine power of a catalytic Janus-sphere micromotor by roughening its surface
Source: Sci Rep. 2018 Mar 15;8:4622. doi: 10.1038/s41598-018-22917-2 (PMC5854611; doi:10.1038/s41598-018-22917-2)
Supplement: Supplementary file 1 — Supplementary Information [file 41598_2018_22917_MOESM1_ESM.pdf]

Supplementary Information

"Improving the engine power of a catalytic Janus-sphere micromotor by roughening its surface"

Brooke W. Longbottom[1] and Stefan A. F. Bon.*[1]

[1]Department of Chemistry, University of Warwick, Gibbet Hill Road, Coventry, CV4 7AL, UK
*s.bon@warwick.ac.uk

Supplementary Video S1 (Accompanied .mp4 video file)

A supplementary video (Supplementary Video S1) shows the transition of a translational to proposed angular propulsion mechanism of a PS-Pt micromotor with large surface deformations (*ld*) dispersed in a 10 vol.% H_2O_2 solution in water. The particle was located near the underlying glass coverslip. This video has been sped up by 4×.
